# Modelling the melting of DNA oligomers with non-inert dangling ends

**DOI:** 10.3389/fmolb.2025.1646428

**Published:** 2025-08-13

**Authors:** Alejandro Soto, Francesco Mambretti, Emanuele Locatelli, Iliya D. Stoev

**Affiliations:** ^1^ Institute of Biological and Chemical Systems - Biological Information Processing, Karlsruhe Institute of Technology, Karlsruhe, Germany; ^2^ Istituto Italiano di Tecnologia, Atomistic Simulations Group, Genoa, Italy; ^3^ Department of Physics and Astronomy, University of Padua, Padua, Italy; ^4^ Istituto Nazionale di Fisica Nucleare (INFN), Sezione di Padova, Padua, Italy

**Keywords:** DNA nanotechnology, melting curves, oxDNA, NUPACK, molecular dynamics, umbrella sampling

## Abstract

In this work, we investigate the dependence of the melting temperature of low-valency DNA constructs on the length of non-inert dangling ends, controlling their sequence composition. We compare two computational models to evaluate their effectiveness and limitations in predicting the melting behavior of DNA oligomers (bivalent linkers) and more complex structures (trivalent nanostars), benchmarking the results against experimental spectroscopic data. Our results suggest that the length of non-inert dangling ends has minimal impact on the melting point of the DNA duplex for the duplexes we studied, informing the future design of DNA supramolecular constructs.

## 1 Introduction

Beyond its primary use as genetic material, DNA has been exploited as a building block for nanoscale technological applications for almost 40 years (Seeman and Sleiman, 2017; Jonoska and Winfree, 2023). DNA nanotechnology employs the same physics and chemistry as DNA’s biological function, using Watson-Crick base-pairing rules of complementarity to achieve high-yield self-assembly of complex structures (Rothemund, 2006; Dey et al., 2021; Liu et al., 2024). As practical applications for DNA-based nanosystems are explored, precise thermodynamic control over the hierarchical self-assembly of complex higher-order structures is paramount (Biffi et al., 2013; Conrad et al., 2019; Dong et al., 2020; Conrad et al., 2022; Stoev et al., 2018; 2020). A deep understanding of structure-property relationships is critical for the advancement of DNA-based nanotechnology, enabling innovative applications, such as environmental monitoring, biomolecular interactions detection and identification of pathogenic biomarkers (Shen et al., 2021; Huang et al., 2021; Can et al., 2025).

The supramolecular assembly of DNA hydrogels and other high-order DNA materials follows a hierarchical, step-by-step strategy, based on complex DNA “building blocks”, constructs that are rather stable at intermediate temperatures and that bind together through short complementary single-stranded sequences called “sticky ends”. This strategy exploits the dependence of the melting temperature on the duplex length: the design allows the building blocks to remain stable in a target thermodynamic region where dynamic formation and breaking of bonds is taken into account, shaping the overall phase diagram (Biffi et al., 2013; Rovigatti et al., 2014; Locatelli et al., 2017; Chen et al., 2020; Abraham et al., 2024).

However, the presence of unpaired bases also influences the stability of the construct. As previously reported (Di Michele et al., 2014), a moderate increase in melting temperature $\left(T_{m}\right)$ is observed upon adding a single unpaired base to a short duplex. Increasing the length $n_{\text{tail}}$ of the resulting dangling end with identical bases, *e.g.*, a series of thymines that acts as a free joint, creates an inert tail and induces a monotonic decrease of $T_{m}$ ; the melting temperature becomes constant for $n_{\text{tail}}$ larger than a few nucleotides, with a plateau value either matching or deviating from the melting temperature of the original duplex $\left(T_{m}^{0}\right)$ , depending on the ionic strength or the detailed design of the construct. This monotonic decrease is driven by electrostatic interactions: after an initial free energy gain due to residual stacking contributions, the presence of residual charges on the single strand may compensate or even overcome the initial rise in $T_{m}$ .

**FIGURE 1:**
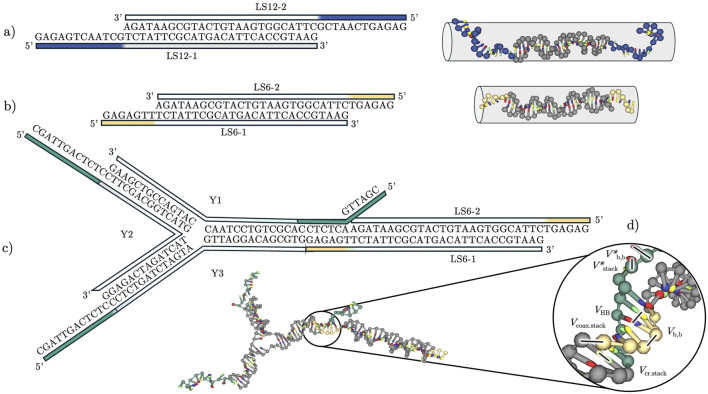
Schematic of the building blocks employed in this study: **(a)** Linker “LS12” with a 12-nucleotide-long sticky end. Linear sequence representation and three-dimensional representation showing the approximate volume the construct occupies. **(b)** Linker “LS6” with a 6-nucleotide-long sticky end. As in **(a)**, we show a linear sequence and three-dimensional representations giving the approximate volume the construct occupies. **(c)** A trivalent nanostar (Y-shape) joined with a double-stranded linker LS6, justifying the rational design of our non-inert sequences, although here we performed simulations and experiments only on the individual building blocks. **(d)** Potential energy contributions included in the oxDNA model, used to calculate intra- and inter-strand interactions.

Here we obtain the melting temperatures as a function of $n_{\text{tail}}$ for similar systems (Figure 1), *i.e.*, simple bivalent or trivalent DNA constructs, with heterogeneous sequences and non-inert tails. The melting temperature reflects the interplay between energy and entropy, serving as a key indicator for assessing the stability and functionality of complex DNA nanostructures. Computational implementations of physical models are often employed to provide estimates of thermodynamic properties, aiming to guide experimental efforts. However, even for relatively simple tasks, widely used software faces challenges related to accurate modelling of experimental conditions and arriving at the required statistics. Here we consider how the melting temperature of a duplex with non-inert tails is affected by $n_{\text{tail}}$ . We tackle the problem experimentally and computationally, using a selection of two popular computational approaches and focusing on the ones of highest practical relevance. We show experimentally that the melting temperature is independent of $n_{\text{tail}}$ ; notably, only one numerical method predicts the experimental trend.

In the following text, we give information on the short DNA constructs employed in this study, specifically bivalent “linkers” (L, Figures 1a,b) and trivalent “Y-shapes” (Y, Figure 1c). We start by briefly presenting experimental methods and theoretical models, NUPACK and oxDNA. We then discuss the experimental ground truth and expose a limitation of NUPACK that, upon inclusion of sticky ends, shows a qualitative discrepancy with experiments, which however can be corrected by using melting curve normalization or a method based on first derivatives to extract melting temperature values (*cf.* Supplementary Figure S4). Instead, results of direct sampling from Molecular Dynamics simulations with the oxDNA model qualitatively reproduce both the literature results (Di Michele et al., 2014) and the experimental data (Can et al., 2025). We conclude with a discussion of our results from a thermodynamic standpoint, critically reviewing and pointing to specific strengths and limitations of each approach.

## 2 Methods

### 2.1 System description

Linkers are bivalent constructs consisting of a 26-base-long double-stranded DNA (dsDNA) core with single-stranded DNA (ssDNA) ends (dangling ends or tails) on both sides. The length of the tails $n_{\text{tail}}$ ranges from 0 to 12 bases at both ends (Figures 1a,b shows $n_{\text{tail}}=6,12$ ); we employ the nomenclature LS X , with 0<X<12 denoting the number of bases in the sticky end). Y-shapes are trivalent nanostars (or trimers) with three interconnected duplex arms that meet in a central core (Figure 1c); each arm of the Y-shape has a 12-base-pair non-inert dangling tail.

Finally, to facilitate comparison with thermodynamic information on shorter oligos, we included in our analysis dimers used in a preceding work (Di Michele et al., 2014) (see SI).

### 2.2 Experimental methods

We ordered the custom-made DNA oligonucleotides in Table 1 from biomers.net GmbH. The oligonucleotides were HPLC-purified, freeze-dried and subsequently dissolved in 10 mM phosphate buffer saline (PBS) supplemented with 100 mM NaCl (pH 7.6), following an established protocol (Stoev et al., 2018; 2020). All oligo concentrations were then measured using NanoDrop 1000 and the hybridisation behaviour verified using polyacrylamide gel electrophoresis and UV-visible spectroscopy. Y-shaped (trivalent) nanostars are formed by the designed single strands Y1, Y2 and Y3, while bivalent DNA linkers are formed by linker strands LS-1 and LS-2. The linkers LS X investigated experimentally are reported in Table 1, where the sticky ends are highlighted with different colours. All samples were stored at 4°C and equilibrated to room temperature prior to spectroscopic experiments.

**TABLE 1:** DNA strands comprising Y-shapes and linkers, with labelled sticky ends (black-green) and hybridising segments between Y1 and Y2, Y1 and Y3, Y2 and Y3 (shades of grey), LSX-1 and LSX-2 (matching colour). All synthesised DNA strands were purified via HPLC before use.

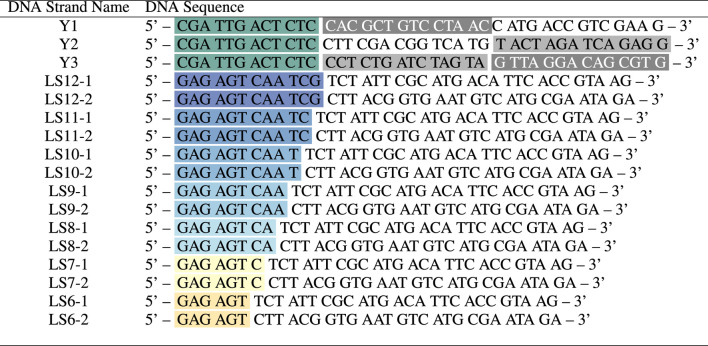

UV-visible absorption spectra were then recorded at a wavelength of 260 nm by Cary 3,500 Multicell Peltier UV-visible spectrophotometer (Agilent Technologies). At this wavelength, we used the differential absorbance of ssDNA and dsDNA to confirm the formation of the Y-shapes and linkers, alongside obtaining information about their melting temperatures. All temperature ramps (heating and cooling) were performed between 25°C and 85°C at a controlled rate of $1{}^{\circ}C\cdot {min}^{-1}$ that minimised hysteretic effects. The oligo concentration in each case was close to 1 μ M, ensuring absorbance values within the specified range of operation of Cary 3,500. The melting temperature $\left(T_{m}\right)$ was determined as the mid-point of the sigmoidal melting curve, where on average half of all hydrogen bonds were broken (heating) or formed (cooling). The uncertainties in the melting temperatures were identified as the differences between the values measured in heating and cooling ramps.

### 2.3 Statistical mechanics approach: NUPACK

The NUPACK tool (Zadeh et al., 2011) was designed to construct and analyse nucleic acid systems, predicting DNA secondary structure from the sequences, *i.e.*, the base-pairs of a set of DNA strands. For each candidate secondary structure, NUPACK algorithms add an empirical estimate of the free energy parameters (Ke et al., 2024), with a strand association penalty energy and stacking contributions (Fornace et al., 2023). The tool allows identification of the Minimum Free Energy (MFE) structure under given conditions, viz., temperature, salt concentration and other thermodynamic parameters. NUPACK advantages include open accessibility, ease of use, and the ability to provide estimates of equilibrium and thermodynamic properties, such as melting profiles, even for systems involving multiple interacting strands (Zadeh et al., 2011). On the other hand, the tool is limited in the properties it can predict for small constructs and may propose highly improbable configurations in the presence of dangling ends (Buterez, 2021). Another issue stems from NUPACK’s inability to distinguish between broken base pairs and single bases belonging to tails, which leads to severe underestimation of bonded fractions at low temperatures for short oligomers. In this analysis, we used the energy parameter set “dna04”, with 100 mM ${[\text{Na}}^{+}$ ] and 1 μ M DNA strand concentration.

### 2.4 Computational approach with oxDNA

OxDNA (Ouldridge et al., 2010a; Šulc et al., 2012; Doye et al., 2013) is a widely used physical model that enables *in-silico* studies of DNA origami and other types of all-DNA fluids, including their self-assembly kinetics. In addition to its effectiveness in reproducing the mechanics and thermodynamics of DNA constructs (Ouldridge et al., 2011; Srinivas et al., 2013; Skoruppa et al., 2018, oxDNA is arguably the most accessible simulation code for DNA, providing introductory material (Sengar et al., 2021; Poppleton et al., 2023), supporting a number of web tools (Poppleton et al., 2021; Bohlin et al., 2022; Poppleton et al., 2020; Suma et al., 2019) and allowing extensions (Kaufhold et al., 2022).

In a nutshell, oxDNA is a top-down, nucleotide-level coarse-grained model that accounts for different potential energy contributions between base pairs (Figure 1d) (Sengar et al., 2021). The oxDNA code supports both Monte Carlo (MC), with native support for Umbrella Sampling (US), and Molecular Dynamics (MD) simulations. Notable for our work is the GPU acceleration of oxDNA, which enables scaling up in simulations of larger systems (Rovigatti et al., 2015).

We performed standard MD simulations, also termed here “unbiased sampling”, and MC simulations with Umbrella Sampling (*cf.* SI). We employed the oxDNA2 model (Snodin et al., 2015), which explicitly includes the effect of screened electrostatic interactions, and set the Na^+^ concentration to S=100 mM, to directly compare with experiments; we performed simulations in bulk using periodic boundary conditions. In MD simulations, we employed an Anderson-like thermostat (“Brownian” in oxDNA), with a damping time of 100 time steps; we set an elementary time step δt=0.005 oxDNA time units. With very short duplexes, we ran M=25 independent replicas of single-molecule simulations, *i.e.*, we simulated a single molecule in a sufficiently large box; for the longer duplexes and the Y-shape, we leveraged oxDNA’s GPU-enabled code, simulating 40 linkers or 30 Y-shapes in a much larger box. In both cases, we started from a low temperature [ T=14° C for oligomers in Table 1, T=52° C for validation of strands from Di Michele et al. (2014)] and let the simulation run for $N_{eq}$ number of steps (*cf.* SI), ensuring that the hydrogen bond contribution to the potential energy reached a plateau. We then sampled the number of hydrogen bonds and computed the fraction of observed hydrogen bonds with respect to a reference maximum value, corresponding to the fully formed duplex state. To construct the melting curve, we then increased the temperature and repeated the procedure, starting from the previous configuration state; we simulated up to T=92° C for oligomers in Table 1 (up to T=68° C for strands from (Di Michele et al., 2014)). In both cases, we applied the bulk extrapolation correction (Ouldridge T. E. et al., 2010); for multiple DNA constructs in the same box, the large size of the box significantly reduced the probability for strands to meet and recombine. Indeed, the latter never occurred in our simulations and hence the results of the two simulation approaches were considered comparable.

## 3 Results and discussion

### 3.1 Validation on reference system

First, we briefly validated our chosen numerical methods on a reference system (Di Michele et al., 2014) (details in the SI). We observed that both unbiased MD and US with oxDNA qualitatively retrieve the reported results. Notably, while we reliably captured a difference in melting temperature $\delta T_{m}≃3{}^{\circ}$ C, associated with the first non-paired base in the dangling end (guanine), we found only incremental changes to the melting curves on further addition of bases to the dangling ends. This we interpreted as a limitation of the oxDNA model and other proposed approaches: US convergence is slow, and classical MD visits uniformly phase space, whereas sampling within the melting region is expected to benefit from advanced sampling techniques. Moreover, short duplexes are highly susceptible to thermal fluctuations close to the melting region, which may push the system towards complete melting. In our scheme, premature melting affects simulations from a certain temperature onwards, as recombination would happen over much longer timescales. For our target bivalent and trivalent constructs, we resorted to unbiased sampling, due to the higher computational cost of the US approach. Validation with NUPACK was considered unsuitable for our short sequences (Buterez, 2021) and was therefore not performed.

### 3.2 Experimental results

**FIGURE 2:**
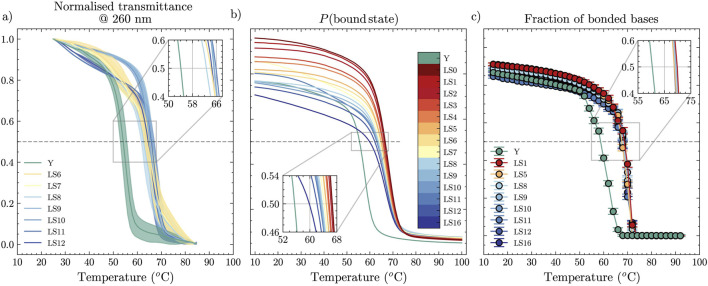
Melting curves as a function of temperature, obtained from **(a)** UV-visible absorbance experiments, **(b)** NUPACK statistical mechanics treatment, and **(c)** oxDNA MD unbiased simulations for dimers with dangling ends of different length and for a Y-shape. Error bars in **(a)** are obtained from the values measured in heating and cooling ramps.

Next, we investigated experimentally through UV-visible spectroscopy a set of duplexes with non-inert tails (Section 2.1), thereby providing ground truth for comparison with the two *in-silico* approaches we selected. We show normalised transmittance at 260 nm in Figure 2a. At this wavelength, single-stranded DNA absorbs more strongly than double-stranded DNA, which yields a sigmoidal curve. The mid-point of the sigmoidal curve–equivalent to the temperature at which the fraction of bonded bases equals 0.5 (a value that closely matches the temperature corresponding to the maximum of the first derivative, as shown in Supplementary Figure S3)–represents a transient dynamic state between a fully assembled and fully dissociated duplex, where on average half of all hydrogen bonds are formed (during cooling) or broken (during heating). Notably, the melting temperature displays little to no variation as we deleted bases from the sticky ends of the double-stranded linkers. However, the Y-shapes display a notably different melting temperature from the linkers, with $T_{m}≃54{}^{\circ}$ C, informing on the hierarchical step-by-step assembly of higher-order systems based on the linking between bivalent linkers and trivalent nanostars.

**FIGURE 3:**
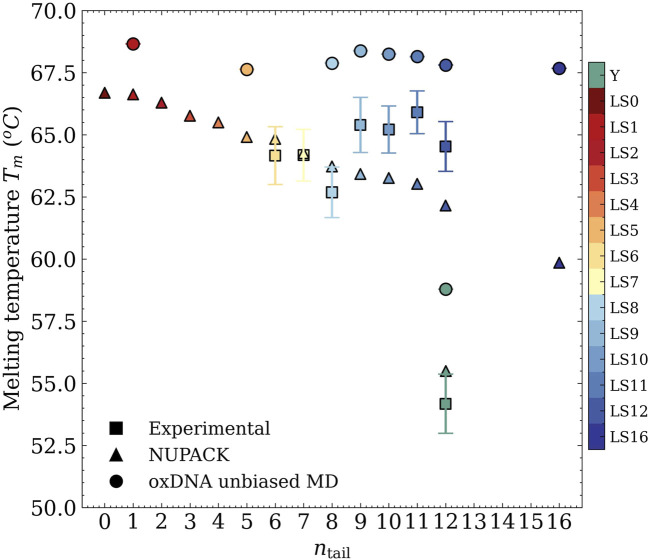
Melting temperature as a function of the length of the sticky ends from UV-visible absorbance experiments (squares), NUPACK statistical treatment (triangles), and oxDNA MD unbiased simulations (circles).

In Figure 3, we included a summary of these experimental melting-temperature results, shown as square symbols, where we indeed observed that $T_{m}$ is roughly constant with $n_{\text{tail}}$ .

### 3.3 NUPACK predictions

For the same oligomers, we computed a prediction of the melting temperature using NUPACK. Figure 2b shows melting curves for different lengths of the dangling ends, $n_{\text{tail}}$ . According to NUPACK, the melting temperature generally shifts down for linkers with longer sticky ends. This prediction seemingly contradicts the experimental evidence for relatively constant melting temperature, since with NUPACK we observe a monotonic decrease with increasing $n_{\text{tail}}$ , without ever reaching a plateau (*cf.* triangles in Figure 3). While NUPACK calculations yield an accurate estimate of $T_{m}$ for short oligos with $n_{\text{tail}}\leq 8$ , they qualitatively fail to reproduce the experimental trends on increasing $n_{\text{tail}}$ . A possible explanation to this observation could be the NUPACK treatment of free bases in the dangling ends, which are included in the total count of non-hybridised bases, precluding the accurate determination of $T_{m}$ . The limitations of NUPACK also originate from the fact that it is a nearest-neighbour model; this suggests lower accuracy in melting temperature estimation, as evidenced by a recent attempt to improve NUPACK’s prediction of secondary structures (Ke et al., 2024).

### 3.4 oxDNA results

Approaching the melting-point determination task using unbiased MD with the oxDNA model leads to the results reported in Figure 2c, where we again provide the curves for different values of $n_{\text{tail}}$ ; error bars represent standard deviation of the fraction of bonded bases, recorded every 1.515 ns in equilibrium simulations. The corresponding $T_{m}$ values as a function of $n_{\text{tail}}$ are represented by circles in Figure 3. While we observe a systematic offset compared to the experimental baseline ( ≈3−6° C), the independence of $T_{m}$ on $n_{\text{tail}}$ is retrieved. This suggests that oxDNA captures more reliably the hybridisation dynamics between DNA filaments with long dangling ends compared to NUPACK. Finally, we note that for relatively long oligomers (40–50 bases), melting events due to statistical fluctuations at $T\ll T_{m}$ are highly improbable. We suggest that the discrepancy between melting temperatures forecast by oxDNA model and those measured in experiments is due to inherent limitations of oxDNA. As NUPACK, it is a nearest-neighbour model, which implies that long-range interactions are not considered, possibly accounting for discrepancies with experiments. Moreover, the oxDNA model is well-suited to reproduce melting properties in bulk, similarly to the SantaLucia model (Santa Lucia and Hicks, 2004); bulk extrapolation then only partially corrects for systems that significantly deviate from the expected bulk conditions.

## 4 Conclusion

We investigated the effect of adding non-inert nucleotides to the sticky end attached to a duplex core in DNA constructs. We compared NUPACK and oxDNA models with an experimental reference; oxDNA was used in the context of Molecular Dynamics simulations, both unbiased and with Umbrella Sampling. In the SI, we summarise additional attempts at using advanced sampling techniques, discussing the challenges and potential pitfalls one encounters along the way. US promotes quicker convergence, but becomes impractical for strands containing even a few tens of bases since the required simulation time increases significantly and it becomes non-trivial to estimate the bias weights. Unbiased MD is cheaper to run and capable of handling larger systems, even with tens of molecules owing to GPU-acceleration; on the other hand, its uniform exploration of states in the melting region implies longer simulation times, since fluctuations become large and the system requires more time to reach steady state. One significant disadvantage of explicit counting of hydrogen bonds in unbiased MD is that it requires careful handling of statistical results by running multiple independent simulations for extended periods. For the long duplexes, the equilibration time was estimated to be approximately $10^{9}$ steps (roughly twice as long close to $T_{m}$ ). The estimation of $T_{m}$ can be improved by running multiple independent simulations and tuning the integration time step. However, oxDNA captures the experimental trend in contrast with NUPACK, showing that $T_{m}$ is independent of $n_{\text{tail}}$ for non-inert bases. In this setting, the thermodynamics of the DNA duplex is largely unaffected by the presence of the dangling ends; this finding complements previous results (Di Michele et al., 2014), considering the effect of inert tails. Moreover, the data support the physical interpretation that sticky ends serve as primary stabilising elements in DNA duplexes. Complex multivalent motifs, such as Y-shapes, exhibit reduced thermal stability due to steric and entropic constraints. As such, this study analyses the advantages and pitfalls of simple, direct approaches for the estimation of the melting temperature *in-silico*, showing that MD unbiased simulations within oxDNA may be used to obtain melting-temperature estimates that are within a few degrees from experimental values. To conclude, the approach outlined here enables the study of linkers with larger $n_{\mathrm{tail}}$ values, or even of more complex structures, such as molecules made by an arbitrary number of Y-shapes and linkers, whose melting curves may otherwise be challenging to tackle with advanced sampling techniques.

## Data Availability

The raw data supporting the conclusions of this article will be made available by the authors, without undue reservation.
