# Supplementary material for: Modelling the melting of DNA oligomers with non-inert dangling ends
Source: Front Mol Biosci. 2025 Aug 13;12:1646428. doi: 10.3389/fmolb.2025.1646428 (PMC12380575; doi:10.3389/fmolb.2025.1646428)
Supplement: Supplementary file 1 [file DataSheet1.pdf]

Supplementary Material

1 SIMULATION DETAILS

1.1 Equilibration in direct sampling of MD runs

Here we provide details on the estimation of a melting curve from unbiased simulations (Fig. S1). As communicated in the main text, we allow the simulation to reach a steady state (Fig. S1a), confirming that the number of hydrogen bonds reaches a constant value in a relatively short time, such that we can obtain an estimate of the number of bonded base pairs and the associated error at the corresponding temperature. We note that these simulations are subjected to out-of-equilibrium sampling, especially close to the melting temperature, since filaments should be allowed to melt and reform, thus affecting the observed number of hydrogen bonds. However, in simulations complete melting and reforming of duplexes occurs on timescales that are inaccessible without introducing a bias, especially at low density. We find that avoiding such states allows us to obtain relatively accurate, albeit still overestimated, results; notably the physics of the system is successfully captured, as highlighted in the main text. Once the average number of hydrogen bonds is computed from simulations, we plot it as a function of temperature (Fig. S1b). Performing the bulk extrapolation (full black squares) improves the determination of the melting temperature, bringing the estimate closer to that obtained in the experimental analogue Ouldridge et al. (2010).

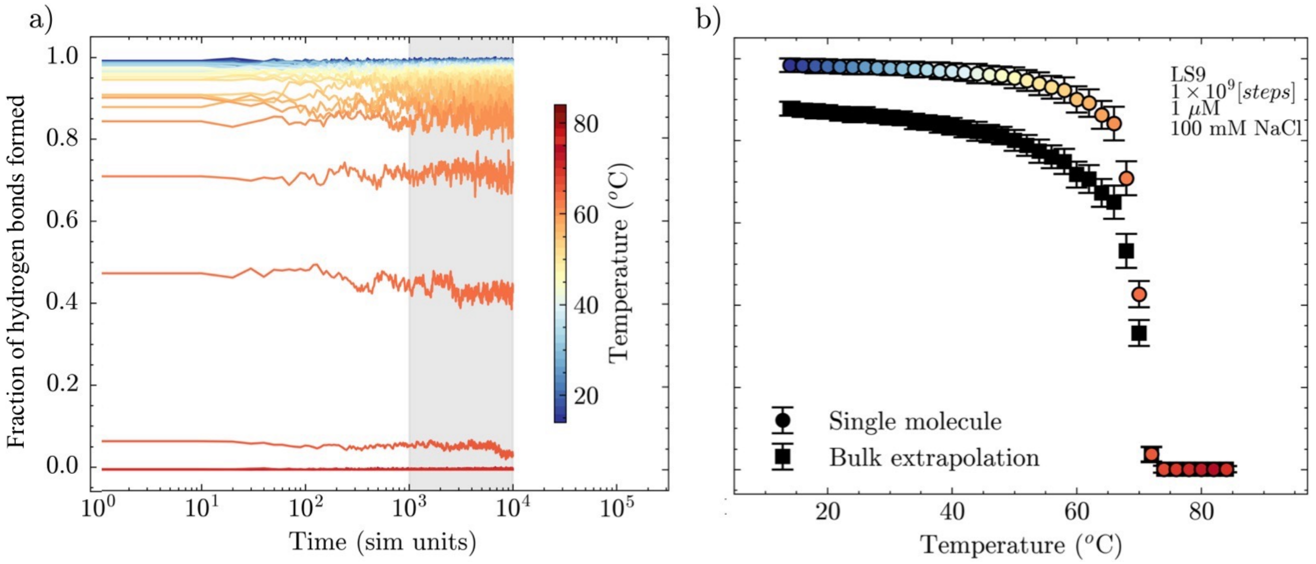

Figure S1: Unbiased melting behaviour. a) Fraction of formed hydrogen bonds as a function of time and temperature; we used this information to assess the steady state of the MD simulations. The shaded area shows the equilibrated data used for the statistical analysis. b) Average fraction of hydrogen bonds as a function of temperature, illustrating the melting behaviour profile for the linker "LS9" at a concentration of 1 μM and salt concentration of 100 mM. Colored circles are the direct estimates, colour code refers to the value of T (colourbar in panel a), black squares result from the applied bulk extrapolation.

1.2 Monte Carlo Umbrella Sampling simulations

As an alternative method, we performed MC simulations with Umbrella Sampling (US) Frenkel and Smit (2023). We employed the Virtual Move Monte Carlo (VMMC) Whitelam and Geissler (2007) to evolve the system and sample between different configurations. The US scheme in the oxDNA code applies a bias to favour or disfavour certain values of a "reaction coordinate", which in this case is the number of native bonded pairs in the system. For a given sequence, we first had to obtain an estimate of the bias weights. To this end, we performed either a long simulation (2×10^9 MC sweeps) or a few shorter ($M = 20$, running for 10^8 MC sweeps) independent simulations, starting from different random initial conditions and evolving at constant bias. We performed these simulations at constant temperature T_0, close to the melting temperature, which we determined using the SantaLucia model (using a Python script, provided with the oxDNA code), so that the system could sample readily states along the reaction coordinate Sengar et al. (2021). From the histogram of the number of bonded pairs, sampled every 10^7 MC sweeps, we estimated the probability $P(n)$ of having n bonded pairs and we constructed the weights $W(n)$ as:

$$W(n) = \exp(-\ln(P(n))), \tag{S1}$$

where n goes from 0, *i.e.* the melted state, to the number of paired base pairs in the duplex $n_{\max}$. Since the free energy is given by $F(n) \simeq -k_B T \ln(P(n))$, we thereby compensated for the differences in free energy between the states along the reaction coordinate. We found this approach to be reliable and easy to implement, although it required performing additional simulations. For the system presented here, one might choose to keep the weights fixed with increasing length of the dangling ends, since the sequence of the central duplex remains the same. However, above one or two dangling bases, recalculation of the weights is necessary to ensure optimal sampling and obtain reliable estimates. We also highlight that, in this case, simulations tend to overestimate the values of the free energy for $n \leq 2$, leading to underestimation of the melting temperature. Rather than increasing the simulation time, we modify the weights pertaining to $n \leq 2$ to correct for this effect. We used the estimated bias to construct the melting curve and compute the melting temperature. The oxDNA code implements histogram reweighting and provides unbiased, reweighted curves along the reaction coordinates, as well as a Python script for estimating the melting temperature, which includes the bulk extrapolation. We performed histogram reweighting in the range $T_0 \pm 15$ K.

2 VALIDATION OF THE METHODS WITH INERT TAILS

We show in Fig. S2 a validation of the melting temperature shift for short DNA duplexes with inert tails, as reported in the literature Di Michele et al. (2014). We contrast the original results, taken from Di Michele et al. (2014) and reported here with red diamond symbols, with the results of oxDNA simulations (unbiased MD and Monte Carlo with Umbrella Sampling) and NUPACK. Results from oxDNA are reported in black circles and pentagons: both datasets showcase the correct trend, characterised by an increase of the melting temperature with the addition of a single unpaired guanine (G) base, followed by a decrease to a plateau. Note that the simplest technique most closely reproduces the reference result. Here, for convenience, we performed $M = 20$ independent parallel simulations at a larger DNA concentration of c = 1 mM and salt concentration of 100 mM to capture the temperature shift. However, here remains necessary to avoid early melting, since even at relatively high concentration recombination occurs on timescales that are inaccessible with unbiased MD simulations. Monte Carlo with Umbrella Sampling results show qualitative agreement with the reference, but quantitatively deviate when $n_{\text{tail}} > 1$. We note that the estimation of the

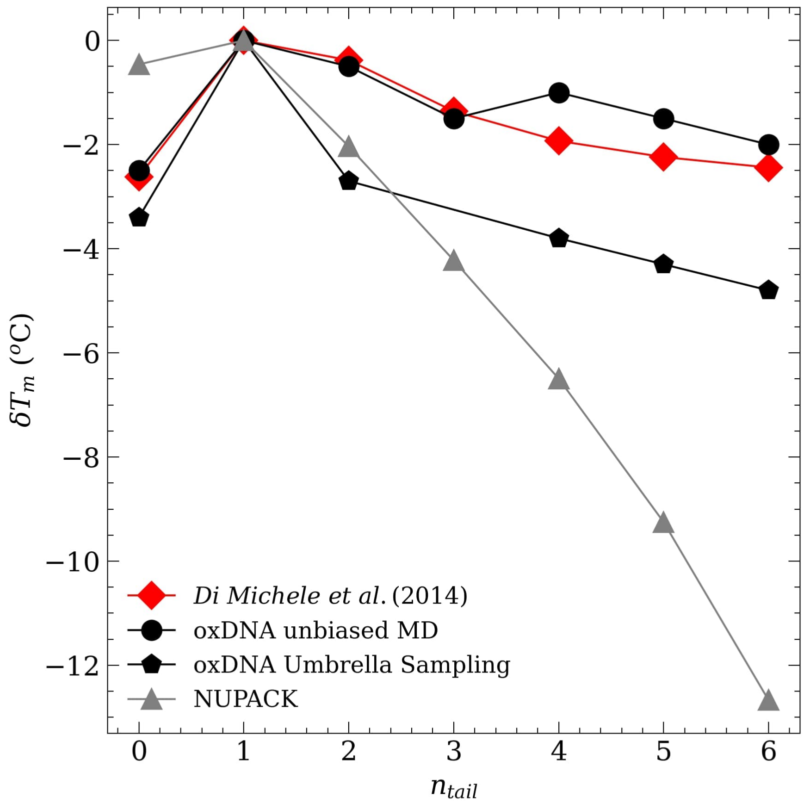

Figure S2: Melting temperature shift as a function of the tail length for the duplexes from Di Michele et al. (2014) with inert tails. Red diamonds refer to the original results, black circles and pentagons refer to results obtained with oxDNA from unbiased MD simulations and Monte Carlo with Umbrella Sampling, respectively, and grey triangles refer to results obtained with NUPACK.

biasing weights becomes increasingly more challenging with increasing $n_{\mathrm{tail}} > 1$; ultimately, Umbrella Sampling convergence is slow and even longer simulations are required. Finally, the results obtained with NUPACK (grey triangles) qualitatively disagree with the reference; as discussed in the main text, NUPACK is limited in reproducing the thermodynamics of short sequences.

3 SUMMARY OF ADDITIONAL ADVANCED SAMPLING TECHNIQUES

We recap here a few advanced sampling techniques that were attempted but did not yield satisfactory results.

- Metadynamics: this advanced sampling technique constructs the bias for each state iteratively in order to compensate for highly improbable states. In this way, one builds the free energy profile over the course of the simulation, from which to compute the melting temperature. We adopted an approach used previously in the literature Kaufhold et al. (2022), which has been implemented in oxDNA. By employing well-tempered Metadynamics, parallel simulations are run with different Metadynamics parameters, allowing to sample different regions of the free energy landscape more efficiently at fixed thermodynamic conditions. A major issue in determining the melting temperature comes from computing the free energy profile at each temperature, thus increasing the computational cost. The choice of the Metadynamics parameters depends on the temperature, which renders the investigation dependent on fine-tuning. Finally, whereas one could try to use the bias to re-weight other observables,

such as the potential energy due to hydrogen bonds, the bias is not directly accessible in the current version of the code, and amending the code would defy the simplicity and ready use of the methods presented in the paper. All in all, while powerful, we find this implementation of Metadynamics not well-suited for the task of computing the melting curve, unless access to other powerful advanced sampling techniques is ensured Zerze et al. (2021).

- Parallel Tempering (PT): known also as Multiple Markov Chain or replica exchange, this advanced sampling technique performs multiple parallel simulations at different temperatures, changing conformations in-between based on a suitable switching scheme. The temperature switch allows the system to sample conformations that may be otherwise rare. Parallel Tempering is not available in oxDNA; while it can be implemented easily by switching conformations with an external code or script, it is available in the simulation code LAMMPS, where oxDNA has been implemented Henrich et al. (2018). Besides requiring additional post-processing, PT ultimately suffers from the same issues encountered with simple unbiased sampling, *i.e.* strand recombination after complete melting requires very long simulations, especially with a relatively large box.

4 FIRST DERIVATIVE DETERMINATION OF THE MELTING TEMPERATURE

The melting temperature (T_m) can be determined with high precision by analyzing the first derivative of the melting curve. Specifically, the derivative of the fraction of unbound bases with respect to temperature (dF/dT) exhibits a maximum at the point of steepest transition. This point corresponds to the inflection point of the original melting profile and is mathematically characterized by the zero-crossing of the second derivative ($d^2F/dT^2 = 0$). In Figure S3, the raw melting data were first smoothened or interpolated, followed by numerical computation of the first and second derivatives. The temperature at which the second derivative vanishes, indicating the maximum slope of the melting curve, is then identified as the melting temperature (T_m).

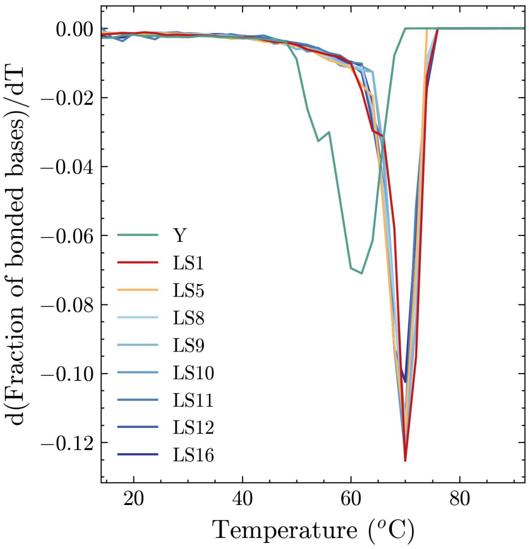

Figure S3: First derivative profiles (dF/dT) of the melting curves for various linker constructs. Each curve represents the temperature-dependent rate of unbinding, highlighting the transition region of DNA melting. The peak of each curve corresponds to the temperature at which the structural transition is most cooperative, allowing accurate determination of the melting temperature (T_m) at the point of maximal slope.

5 NORMALIZATION OF NUPACK RESULTS

The normalization of the results from the theoretical estimation was performed as a potential strategy to correct the trend observed in the experimental data. However, this type of normalization should be treated with caution, as NUPACK estimates rely on empirical parameters that may be invalidated or misrepresented when the data are excessively post-treated.

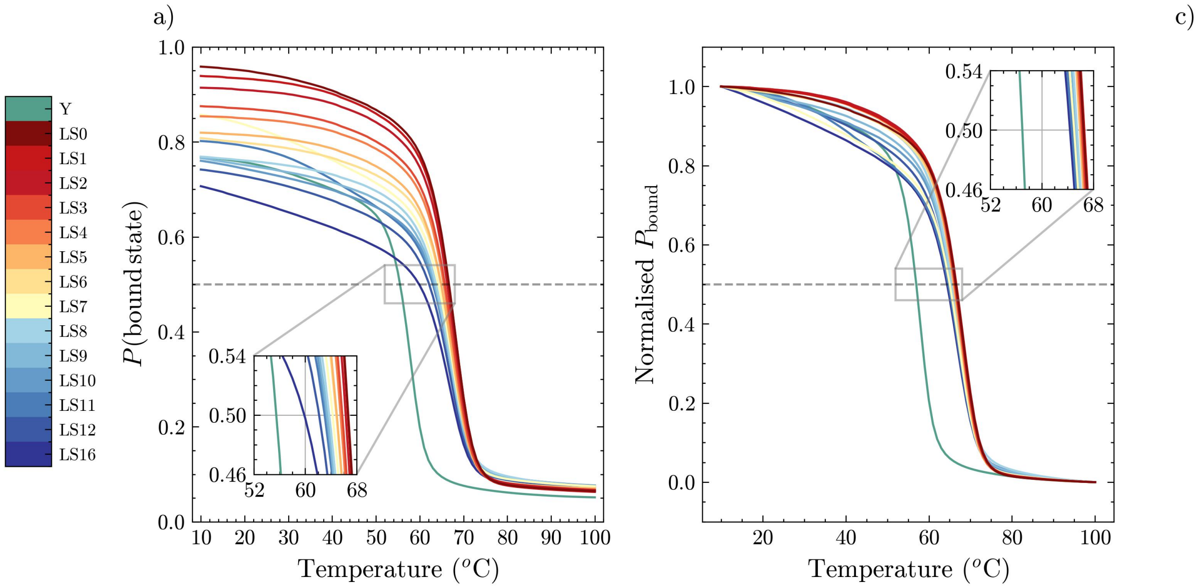

Figure S4: NUPACK output of the equilibrium bound state probability for DNA linkers. (a) Fraction of bases in the bound state as a function of temperature, P(bound state), directly obtained from NUPACK calculations for a series of DNA linkers with varying sticky-end lengths. (b) Normalized version of the same profiles, $P_{\text{bound}}^{\text{norm}}$, to highlight the sharpness of the melting transition and facilitate comparison across linkers. Insets show zoom-ins around the transition region ($P \approx 0.5$). The vertical color bar indicates the identity of each linker.

REFERENCES

Di Michele, L., Mognetti, B. M., Yanagishima, T., Varilly, P., Ruff, Z., Frenkel, D., et al. (2014). Effect of inert tails on the thermodynamics of dna hybridization. *Journal of the American Chemical Society* 136, 6538–6541

Frenkel, D. and Smit, B. (2023). *Understanding molecular simulation: from algorithms to applications* (Elsevier)

Henrich, O., Gutiérrez Fosado, Y. A., Curk, T., and Ouldridge, T. E. (2018). Coarse-grained simulation of dna using lammps. *The European Physical Journal E* 41, 57. doi:10.1140/epje/i2018-11669-8

Kaufhold, W. T., Pfeifer, W., Castro, C. E., and Di Michele, L. (2022). Probing the mechanical properties of dna nanostructures with metadynamics. *ACS nano* 16, 8784–8797

Ouldridge, T. E., Louis, A. A., and Doye, J. P. (2010). Extracting bulk properties of self-assembling systems from small simulations. *Journal of Physics: Condensed Matter* 22, 104102

Sengar, A., Ouldridge, T. E., Henrich, O., Rovigatti, L., and Šulc, P. (2021). A primer on the oxdna model of dna: when to use it, how to simulate it and how to interpret the results. *Frontiers in Molecular Biosciences* 8, 693710

Whitelam, S. and Geissler, P. L. (2007). Avoiding unphysical kinetic traps in monte carlo simulations of strongly attractive particles. *The Journal of chemical physics* 127

Zerze, G. H., Stillinger, F. H., and Debenedetti, P. G. (2021). Thermodynamics of dna hybridization from atomistic simulations. *The Journal of Physical Chemistry B* 125, 771–779
